# Unravelling the interplay of sphingolipids and TGF-β signaling in the human corneal stroma

**DOI:** 10.1371/journal.pone.0182390

**Published:** 2017-08-14

**Authors:** Sarah E. Nicholas, Tyler G. Rowsey, Shrestha Priyadarsini, Nawajes A. Mandal, Dimitrios Karamichos

**Affiliations:** 1 Department of Ophthalmology/ Dean McGee Eye Institute, University of Oklahoma Health Sciences Center, Oklahoma City, Oklahoma, United States of America; 2 University of Oklahoma College of Medicine, Oklahoma City, Oklahoma, United States of America; 3 Department of Ophthalmology /University of Tennessee Health Science Center, Memphis, Tennessee, United States of America; 4 Department of Anatomy and Neurobiology /University of Tennessee Health Science Center, Memphis, Tennessee, United States of America; 5 Department of Cell Biology/ University of Oklahoma Health Sciences Center, Oklahoma City, Oklahoma, United States of America; Medical University of South Carolina, UNITED STATES

## Abstract

**Purpose:**

To delineate the role of Sphingolipids (SPLs) in the human cornea and their cross-talks with transforming growth factor beta (TGF-β) in order to develop novel, non-invasive therapies.

**Methods:**

Human corneal fibroblasts (HCFs) were harvested from healthy donors, stimulated with Vitamin C to promote extracellular matrix assembly, treated with exogenous sphingosine-1-phosphate (S1P) or sphingosine kinase inhibitor 2 (SPHK I_2_) and isolated after 4 weeks for further analysis.

**Results:**

Data showed that S1P led to a significant decrease in cellular migration where SPHK I_2_ just delayed it for 24h. Significant modulation of the sphingolipid pathway was also noted. Sphingosine kinase-1 (SphK1) was significantly downregulated upon exogenous stimulation with S1P at a concentration of 5μM and Sphingosine kinase-2 (SphK2) was also significantly downregulated at concentrations of 0.01μM, 0.1μM, and 5μM; whereas no effects were observed upon stimulation with SPHK I_2._ S1PR3 was significantly downregulated by 0.1μM and 5μM S1P and upregulated by 5μM and 10μM SPHK I_2_. Furthermore, both S1P and SPHK I_2_ regulated corneal fibrosis markers such as alpha-smooth muscle actin, collagen I, III, and V. We also investigated the interplay between two TGF-β isoforms and S1P/SPHK I_2_ treatments and found that TGF-β1 and TGF-β3 were both significantly upregulated with the 0.1μM S1P but were significantly downregulated with the 5μM S1P concentration. When TGF-β1 was compared directly to TGF-β3 expression, we observed that TGF-β3 was significantly downregulated compared to TGF-β1 in the 5μM concentration of S1P. No changes were observed upon SPHK I_2_ treatment.

**Conclusion:**

Our study delineates the role of sphingolipids in the human cornea and highlights their different activities based on the cell/tissue type.

## Introduction

Corneal fibrosis, or corneal scarring, is characterized by the emergence of myofibroblasts and excessive deposition of extracellular matrix components (ECM) [1–4]. This leaves the cornea opaque and can result in partial or complete vision loss [5–9]. Currently, more than 10 million people worldwide are blind because of corneal scarring and approximately 100 million suffer from impaired vision. The mechanics of fibrosis have been studied for years, but there are currently no available drugs for scarring treatment. Recently the role of sphingolipids (SPLs) has been linked to fibrosis in a variety of tissues and organs [10–13].

Bioactive SPLs most notably Sphingosine-1-phosphate and ceramide (Cer), are now recognized to be important mediators of many basic cellular processes such as cell to cell interactions, cell migration, proliferation, survival, contraction, and gene expression [10]. The impact of SPLs in human diseases associated with inflammation, neovascularization, tumorigenesis, and diabetes have been recognized but are still understudied [14–19]. S1P has been established as a “growth-like” factor due to its pleiotropic nature and therefore, by virtue of their ability to regulate diverse cellular processes, there has been great recent interest in the ability to regulate tissue fibrosis in various organ systems using S1P and/or Cer (Roger A. Sabbadini, 2010). S1P has been studied more extensively than Cer in regards to tissue fibrosis. Studies include numerous organ systems, such as lungs [20], skin [21–24], liver [25–29], heart [30,31], and eye [11,32–35].

Interestingly, the role of S1P in fibrosis is somewhat controversial. It was originally characterized as a powerful stimulator of fibroblast proliferation in Swiss 3T3 cells [36]. S1P has also been shown to inhibit the proliferation of hepatic myofibroblasts [37] in human epidermal keratinocytes [38]. In the lungs, S1P signaling through sphingosine-1-phosphate receptor 1 S1P_1_ appears to protect against the development of fibrosis. Conversely, S1P appears to promote fibrosis in other organ systems [10] (skin, liver, heart, retina) likely through activation of TGF-β signaling pathways and/or by promoting fibroblast migration.

Surprisingly, very little is known about the role of SPLs in the human cornea and the mechanisms of corneal fibrosis. In fact, there are only two reports that showed the presence of *Sphingosine kinase-1 (*SphK1), *Sphingosine kinase-2* (SphK2), S1P_1-3,5_ receptor proteins [11] and mRNA [39] in cultured human primary corneal fibroblasts. Expression of S1P receptor’s mRNA have also been noted by [40], in cultured corneal epithelial cells mimicking wound healing responses *in vivo*.

In this study, using our established 3D *in vitro* model, for the first time we investigated the molecular involvement of S1P in human corneal fibroblasts (HCFs) and the interplay between S1P and TGF-β isoforms.We observed that S1P had prominent effects on cell migration, fibrotic markers, and ECM assembly in HCFs, therefore delineating the role of SPLs in the human cornea might pave the way for novel therapeutic agents designed to reduce or reverse fibrosis.

## Materials and methods

### Ethics and inclusion criteria

Study followed the tenets of the Declaration of Helsinki. Corneal samples were obtained from the Dean McGee Eye Institute Clinic. The IRB at the Oklahoma University Health Sciences Centre was notified of our receipt of these tissues, and has determined that this project does not meet the definition of human subject research. The tissue used included corneal samples from donors with no history of ocular trauma or systemic diseases.

### Human corneal fibroblast cultures

Primary HCFs were isolated and cultured from healthy human corneas obtained from the National Disease Research Interchange (NDRI, Philadelphia, PA). All research adhered to the tenets of the Declaration of Helsinki. Briefly, corneal epithelium and endothelium were scrapped and removed from donor corneas. Stromal tissue cut into 2x2mm pieces was placed into T25 flasks and allowed to adhere. Explants were cultured with Eagle’s Minimum Essential Medium (EMEM: ATCC; Manassas, VA) containing 10% fetal bovine serum (FBS: Atlantic Biological’s; Lawrenceville, CA) and 1% antibiotic (Gibco^®^ Antibiotic-Antimycotic: Life Technologies; Grand Island, NY). The cells were allowed to grow to 100% confluence at 37°C, 5% CO_2_. Passages 5–7 were used throughout the experiments.

HCFs were plated on six-well size polycarbonate membrane inserts with 0.4-μm pores (Transwell; Corning Costar; Charlotte, NC) at a density of 1×10^6^ cells/ml [41–44].The cells were cultured in EMEM with 10% FBS and stimulated with a stable Vitamin C derivative (0.5mM 2-*O*-α-D-glucopyranosyl-L-ascorbic acid: American Custom Chemicals Corporation; San Diego, CA). Cultures were grown for 4 weeks before further processing. Cultures without any treatment served as the controls (C), and fresh media were supplied every other day for the duration of the experiment. Three different groups were tested: (1) *Control (C)*: EMEM+FBS+VitC (construct medium); (2) *S1P*: construct medium+S1P at 0.01μM, 0.1μM, or 5μM [13,45]; and (3) SPHK I_2_: construct medium+SPHK I_2_ at 2μM, 5μM, and 10μM. A stock solution of S1P (Avanti Polar Lipids; Alabaster, AL) was made at a concentration of 125μM for all S1P treatments by dissolving S1P powder in 4mg/ml of BSA in water at 37°C in a glass vessel. SPHK I_2_ (Cayman Chemicals, USA) is a selective inhibitor of SphK1 [46] and a stock solution was made at a concentration of 5mM by dissolving the powder in DMSO. All experimental groups were repeated at least three times before processed for protein expression using Western Blot analysis.

### RNA quantification and real-time PCR

RNA was extracted from constructs using an RNA mini extraction kit (Ambion TRIzol® Plus RNA Purification Kit: Life technologies, Carlsbad, CA) followed by cDNA synthesis using SuperScript™ III First-Strand Synthesis SuperMix (Invitrogen; Carlsbad, CA) according to the manufacturer’s protocol. The TaqMan gene expression assays (Applied Biosystems, Foster City) GAPDH (Hs99999905_m1) and 18S (Hs99999901_s1) were used as endogenous controls while S1PR1 (Hs01922614_s1) and S1PR3 (Hs00245464_s1) were the study probes. cDNA amplification was performed using the StepOnePlus^TM^ real-time PCR system (Life Technologies; Carlsbad, CA).

### Protein quantification and western blot

Western blot analyses were performed on all constructs and their protein concentrations and purities were determined via Pierce™ BCA Protein Assay (ThermoFisher Scientific; Rockford,IL). Precast Novex 4–20% Tris-Glycine Mini Gels (Life Technologies; Carlsbad, CA) were used for gel electrophoresis and transferred onto nitrocellulose membranes (Bio-Rad Laboratories, Inc; Hercules, CA). After a one hour incubation in a 5% BSA blocking solution (ThermoScientific; Rockford, IL), the membranes were incubated with primary rabbit polyclonal antibodies: anti-TGF-β1 (Abcam; Cambridge, MA), anti-TGF β3 (Abcam; Cambridge, MA), anti-SphK1 (Abcam; Cambridge, MA), anti-SphK2 (Abcam; Cambridge, MA), anti-EDG3 (S1PR3; Abcam; Cambridge, MA), anti-Collagen I (Abcam; Cambridge, MA), anti-Collagen III (Abcam; Cambridge, MA), anti-Collagen V (Abcam; Cambridge, MA), and anti-alpha smooth muscle Actin (Abcam; Cambridge, MA) at 1:1000 dilutions overnight at 4°C. Subsequently, the membranes were washed and incubated with a secondary antibody (Alexa Flour® 568 Donkey anti-Rabbit, IgG (H^+^L); Life Technologies; Carlsbad, CA) at 1:2000 dilutions for one hour. The UVP imaging system (VisionWorks™LS Image Acquisition & Analysis Software; Cambridge, UK) was used for signal detection. The results were analyzed by normalizing the values to that of the house keeping antibody, anti-beta Actin (Abcam; Cambridge, MA) expression, and the fold expression was plotted.

### Cellular migration

Cell migration was measured using an *in vitro* 2D scratch assay method [47] where HCFs were seeded into 6-well plates at a density of 1×10^6^ cells/ml and cultured in the following: (1) Control (C): construct medium; (2) *S1P*: construct medium+S1P at 0.01μM, 0.1μM, or 5μM; and (3) SPHK I_2_: construct medium+ SPHK I_2_ at 2μM, 5μM, and 10μM. After a 24-hour incubation period, allowing the cells to adhere, a scratch was administered through the confluent cell layer using a pipette tip. An AmScope IN200TA-M Digital Long Working Distance Inverted Trinocular Microscope (Irvine, CA) was used to capture images of each scratch wound which was repeated at intervals of 0, 4, 24, and 48 hours to monitor wound closure. Cell migration was measured and quantified using ImageJ software.

### Statistical analysis

All experiments were repeated at least 3 times and the statistical significance was performed by ANOVA and/or non-parametric *t* test analysis, where p<0.05 was considered to be statistically significant. Graph Pad Prism 6 software was used for all statistical analysis.

## Results

### Effects of S1P and SPHK I2 on cellular migration

S1P is formed by active SphK [48,49] and signals through S1P receptors 1–5 which preferentially couple to different downstream pathways. The downstream pathways of S1PR1-S1PR5 are reportedly overlapping yet functionally oppositional; with various intracellular targets that are known to engage in signal processing [50]. S1P is known to stimulate cell proliferation and migration in human aortic endothelial cells [51], murine satellite cells [52], WiT49 cells [53], human thyroid cancer cells [54], and many other cell types and can be inhibited by SPHK I_2_ which is a selective inhibitor of SphK1 with anti-proliferative capacity [46]. Studies have shown that corneal fibroblasts and myofibroblasts modulate important aspects of cell behavior such as adhesion, migration, and differentiation [55,56].

**Fig 1:**
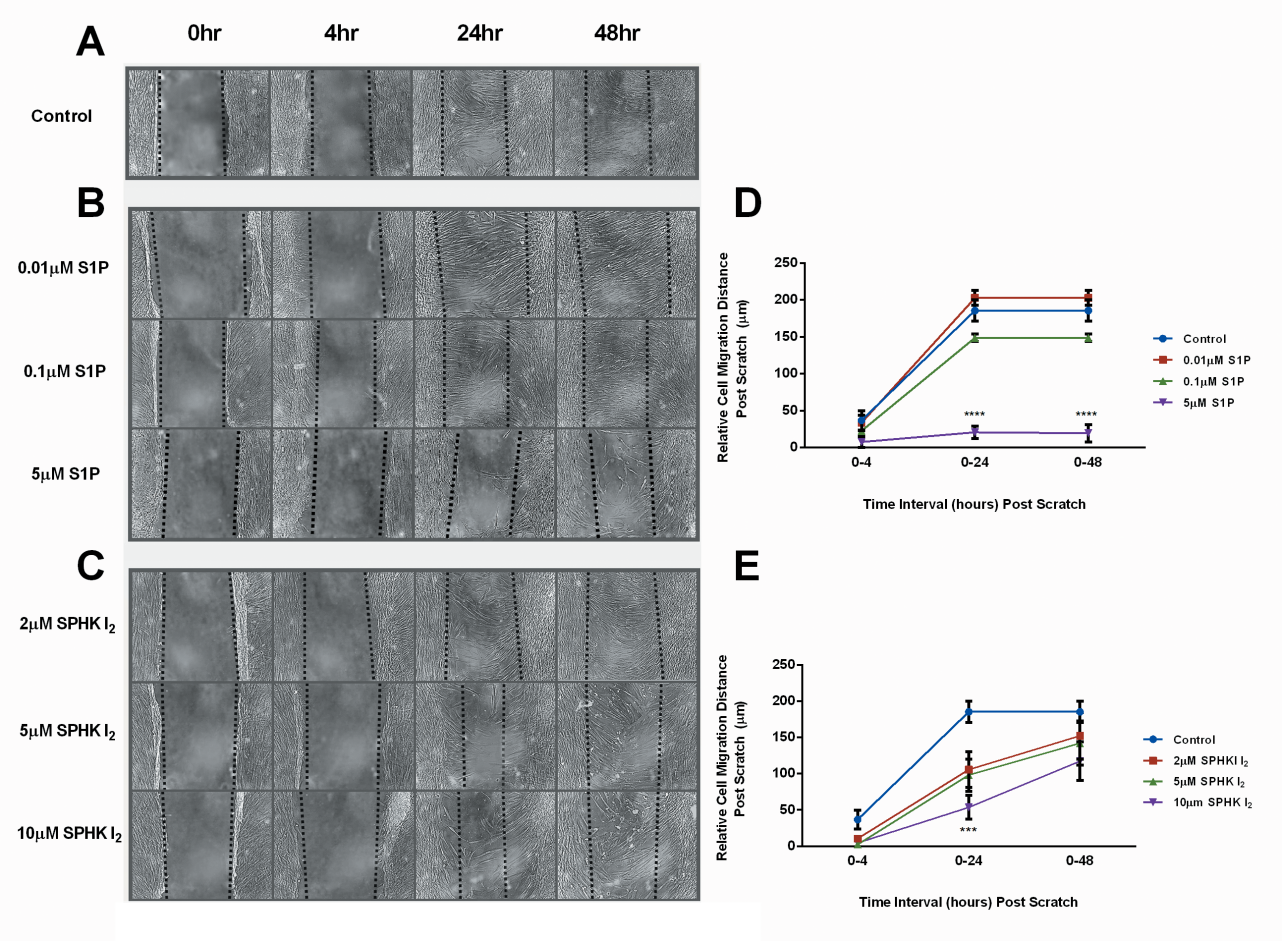
Effects of S1P and SPHK I_2_ on cellular migration/Scratch wound healing assay. (A-C) HCFs were scratched and the relative cell migration percentage was quantified at 0, 4, 24, and 48 hour time points. (D) Scratch assay quantification of S1P stimulated HCFs showing significant decrease (p ≤ 0.0001) in cell migration distance at 24 and 48 hour time points compared to controls (A). (E) Scratch assay quantification of SPHK I_2_ stimulated HCFs showing significant decrease (p ≤ 0.001) in cell migration distance at the 24 hour time point compared to controls (A). (A-C) Dotted lines indicate cell fronts or origin location of scratch. A one-way ANOVA was performed and p ≤ 0.05 considered statistically significant. * represents p ≤ 0.05, ** represents p ≤ 0.01, *** represents p ≤ 0.001, **** represents p ≤ 0.0001.

We investigated and quantified cell migratory pattern for HCFs when stimulated with both S1P (Fig 1B) and SPHK I_2_ (Fig 1C), at various concentrations compared to controls (Fig 1A). Using an *in vitro* scratch assay model we observed decreased cellular migration with all S1P concentrations (Fig 1D). The highest concentration tested here, 5μM S1P, showed the most severe and significant decrease at both 24 and 48h time points (p≤0.0001). At 24h and 48h, cellular migration with 5μM S1P stimulated cells only closed 14% of the wound when compared to 100% with Controls.

Similar pattern was observed with SPHK I_2_ stimulated cells (Fig 1E). At 24h, controls had closed the wound (100%) where cells stimulated with 10uM SPHK I_2_ only managed to close 44% (p≤0.01) of the wound indicating severe interruption of cellular migration patterns by SPHK I_2_ (Fig 1C), similar to S1P (Fig 1B), however, at the 48h time point the wound had fully closed.

### Effects of S1P and SphKI2 on sphingolipid-related pathways

We investigated the roles of S1P and SPHK I_2_ in HCFs, using our 3D *in vitro* model.

**Fig 2:**
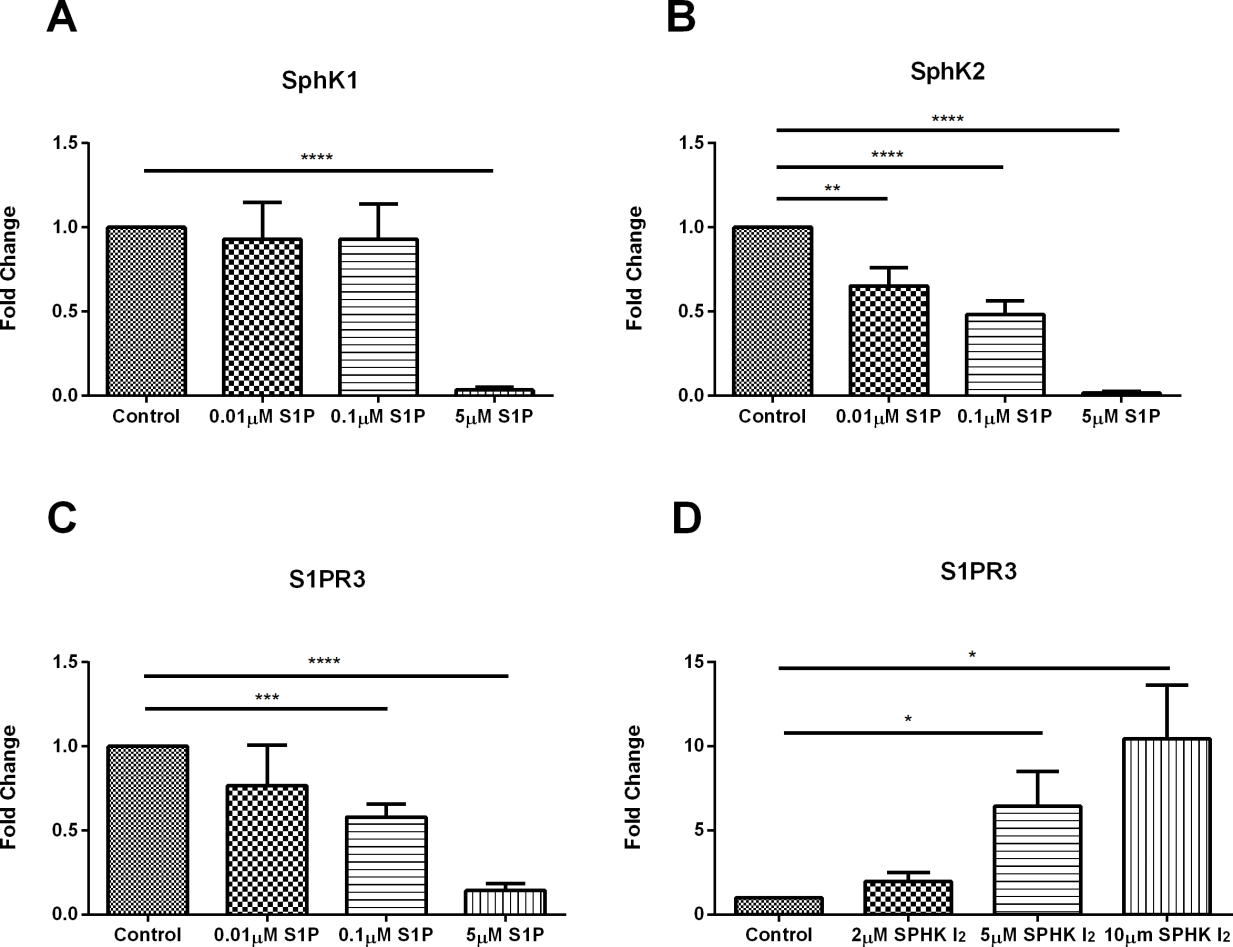
Effects of S1P and SPHK I_2_ on sphingolipid-related pathways/Quantification of western blots of HCF cell lysates following four week treatments with S1P or SPHK I_2_. (A) Treatment with 5μM concentration of S1P on HCF 3D constructs led to significant downregulation (p ≤ 0.0001) of SphK1 when compared to control HCFs. (B) Treatment with 0.01μM, 0.1μM, and 5μM concentrations of S1P on HCF 3D constructs led to significant downregulation (p ≤ 0.01, p ≤ 0.0001, p ≤ 0.0001) of SphK2 when compared to controls. (C) Treatment with 0.1μM and 5μM concentration of S1P on HCF 3D constructs led to significant downregulation (p ≤ 0.001 and p ≤ 0.0001) of S1PR3 when compared to controls. (D) Treatment with 2μM, 5μM, and 10μM concentrations of SPHK I_2_ on HCF 3D constructs led to significant upregulation (p ≤ 0.05) of S1PR3 when compared to controls. A one-way ANOVA was performed and p ≤ 0.05 considered statistically significant. * represents p ≤ 0.05, ** represents p ≤ 0.01, *** represents p ≤ 0.001, **** represents p ≤ 0.0001.

Exogenous S1P stimulation led to significant downregulation of SphK1 protein expression (Fig 2A; p≤ 0.0001; S1 Fig) at 5μM concentration whereas of SphK2 was significantly downregulated at 0.01μM (Fig 2B; p≤0.01;S1 Fig), 0.1μM (Fig 2B; p≤0.0001; S1 Fig), and 5μM (Fig 2B; p≤0.0001; S1 Fig) concentrations. Interestingly, exogenous SPHK I_2_ had no significant effects on SphK1 or SphK2 across all the concentrations tested here (2μM, 5μM, and 10μM).

In addition, Fig 2C (S1 Fig) shows significant downregulation of sphingosine-1-phosphate receptor 3 (S1PR3) protein expression. S1PR3 is one of five G-protein-coupled receptors that bind S1P and transactivates downstream signaling pathways. S1PR3 downregulation was observed following stimulation with S1P at concentrations of 0.1μM (p≤0.0001) and 5μM (p≤0.0001), whereas in Fig 2D (S1 Fig) we detected significant upregulation of S1PR3 upon stimulation with 5μM (p≤0.01) and 10μM (p≤0.01) concentrations of SPHK I_2_.

### Effects of S1P and SPHK I2 on fibrosis (assembly)

In the healthy human cornea, Collagen I and V are the dominant collagens, however following and injury or trauma, Collagen III expression is massively upregulated leading to eventual fibrosis. An almost concurrent event to Collagen III upregulation is the stimulation and differentiation of the resident keratocytes into myofibroblasts. These cells are known to express high level of α-SMA. α-SMA it is therefore a well-known marker for corneal fibrosis.

**Fig 3:**
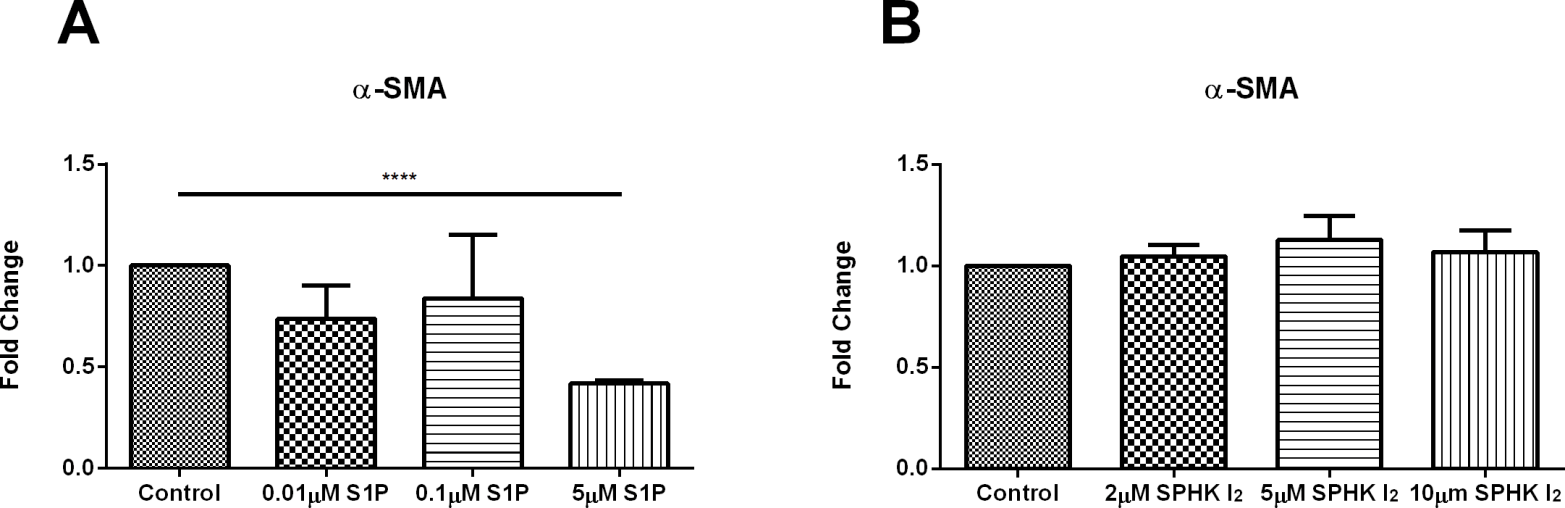
Effects of S1P and SPHK I_2_ on fibrosis/Quantification of western blots of HCF cell lysates following four week treatments with S1P or SPHK I_2_. (A) Treatment with 5μM concentration of S1P on HCF 3D constructs led to significant downregulation (p ≤ 0.0001) of α-SMA when compared to control HCFs. (B) Treatment with 2μM, 5μM, and 10μM concentrations of SPHK I_2_ on HCF 3D constructs had no effect on α-SMA when compared to control HCFs. A one-way ANOVA was performed and p ≤ 0.05 considered statistically significant. * represents p ≤ 0.05, ** represents p ≤ 0.01, *** represents p ≤ 0.001, **** represents p ≤ 0.0001.

We determined the effects of both S1P and SPHK I_2_ as they relate to corneal fibrosis and fibrotic markers in HCFs. In Fig 3A (S2 Fig) shows significant downregulation was observed (p≤0.0001) in α-SMA protein expression upon stimulation with only 5μM S1P with no changes observed when constructs were stimulated with SPHK I_2_ (Fig 3B; S2 Fig).

**Fig 4:**
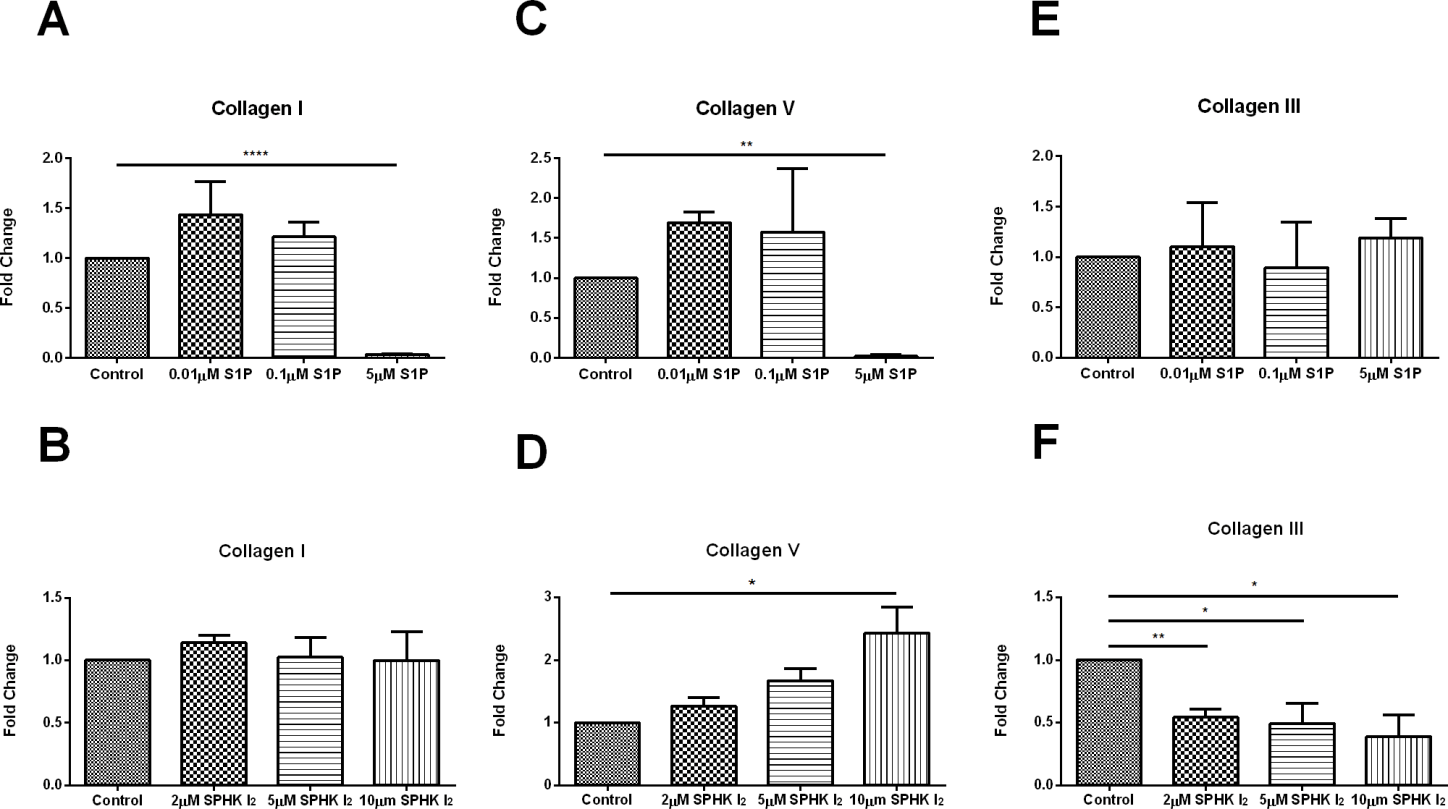
Effects of S1P and SPHK I_2_ on fibrosis (assembly)/Quantification of western blots of HCF cell lysates following four week treatments with S1P or SPHK I_2_. (A) Treatment with 5μM concentration of S1P on HCF 3D constructs led to significant downregulation (p ≤ 0.0001) of Collagen I when compared to control HCFs. (B) Treatment with 2μM, 5μM, and 10μM concentrations of SPHK I_2_ on HCF 3D constructs had no effect on Collagen I when compared to control HCFs. (C) Treatment with 5μM concentration of S1P on HCF 3D constructs led to significant downregulation (p ≤ 0.0001) of Collagen V when compared to control HCFs. (D) Treatment with 10μM concentration of SPHK I_2_ on HCF 3D constructs led to significant upregulation (p ≤ 0.05) of Collagen V when compared to control HCFs. (E) Treatment with 0.01μM, 0.1μM, and 5μM concentrations of S1P on HCF 3D constructs had no effect on Collagen III when compared to control HCFs. (F) Treatment with 2μM, 5μM, and 10μM concentrations of SPHK I_2_ on HCF 3D constructs led to significant upregulation (p ≤ 0.01, p ≤ 0.05, and p ≤ 0.05) of Collagen III when compared to control HCFs. A one-way ANOVA was performed and p ≤ 0.05 considered statistically significant. * represents p ≤ 0.05, ** represents p ≤ 0.01, *** represents p ≤ 0.001, **** represents p ≤ 0.0001.

Collagen I was significantly downregulated (Fig 4A; p≤0.0001;S3 Fig) when constructs were stimulated with 5μM S1P but no change when constructs were stimulated with SPHK I_2_ (Fig 4B;S3 Fig). Collagen V was significantly downregulated (Fig 4C; p≤0.0001; S3 Fig) upon stimulation with 5μM S1P and significantly upregulated (Fig 4D; p≤0.01;S3 Fig) with 10μM SPHK I_2_ stimulation.

We observed no significant changes of collagen III upon S1P stimulation (Fig 4E; S3 Fig while SPHK I_2_ exogenous stimulation led to significant downregulation (2μM; p≤0.001, 5μM; p≤0.01, and 10μM; p<0.01) across all the concentrations tested here (Fig 4F;S3 Fig).

**Fig 5:**
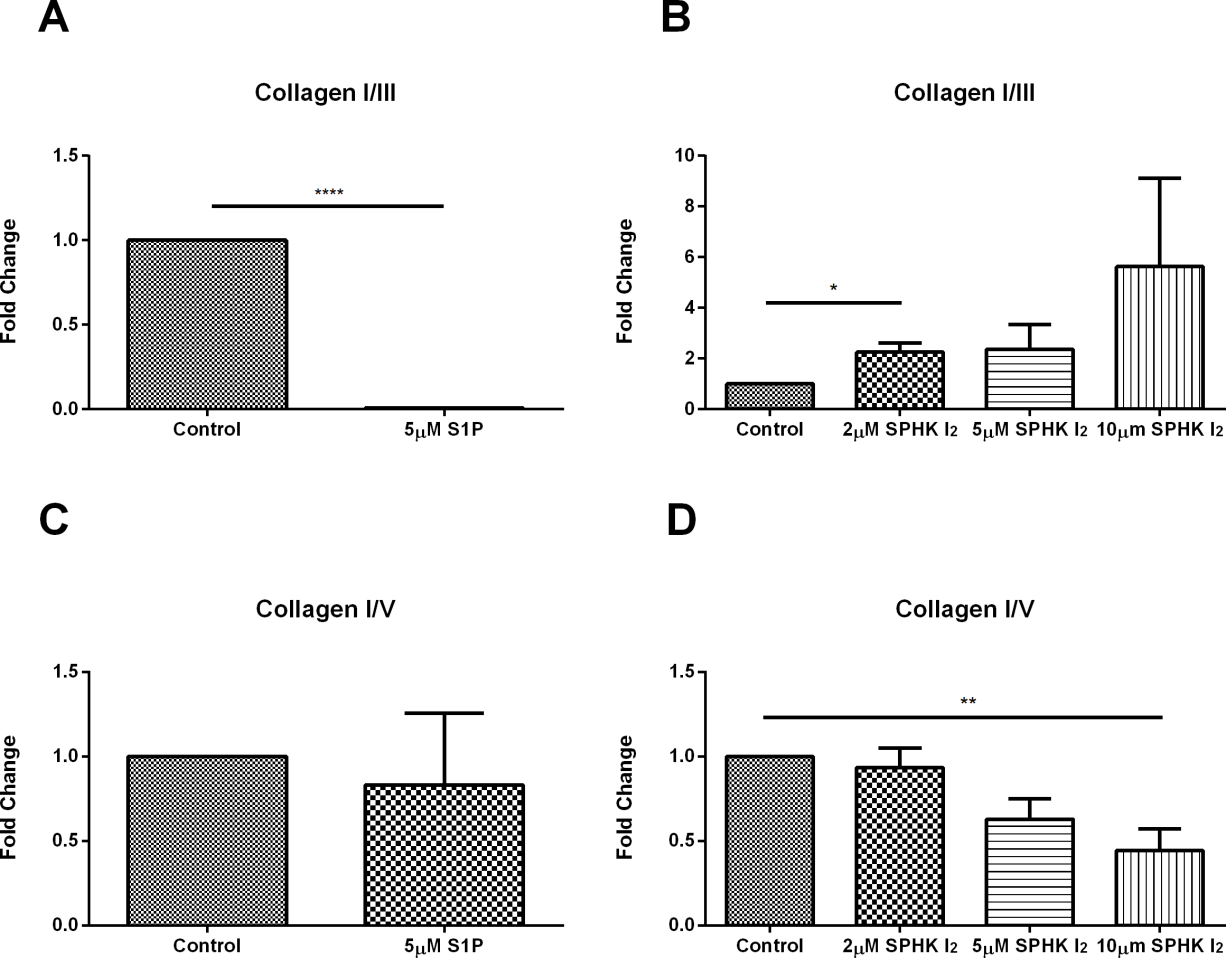
Effects of S1P and SPHK I_2_ on fibrosis (assembly)/Quantification of western blots of HCF cell lysates following four week treatments with S1P or SPHK I_2_. (A) Treatment with 5μM concentration of S1P on HCF 3D constructs led to significant downregulation (p ≤ 0.0001) of Collagen I/III when compared to control HCFs. (B) Treatment with 2μM concentration of SPHK I_2_ on HCF 3D constructs led to significant upregulation (p ≤ 0.05) on Collagen I/III when compared to control HCFs with an upward trend observed with 5μM and 10μM treatment concentrations. (C) Treatment with 5μM concentration of S1P on HCF 3D constructs had no effect on Collagen I/V when compared to control HCFs. (D) Treatment with 10μM concentration of SPHK I_2_ on HCF 3D constructs led to significant downregulation (p ≤ 0.01) of Collagen I/V when compared to control HCFs. A one-way ANOVA was performed and p ≤ 0.05 considered statistically significant. * represents p ≤ 0.05, ** represents p ≤ 0.01, *** represents p ≤ 0.001, **** represents p ≤ 0.0001.

When Collagen I/III ratio was analyzed, stimulation with 5μM S1P led to significant downregulation (Fig 5A; p≤0.0001) where significant upregulation (Fig 5B; p<0.01) was observed upon stimulation with only 2μM SPHK I_2_ and an upward trend in the higher concentrations. Collagen I/V ratio was unaffected following S1P stimulation (Fig 5C) where a downward trend was observed through the SPHK I_2_ treatment concentration series with significance (Fig 5D; p<0.001) observed in the 10μM concentration only.

### S1P and SPHK I2 signaling interactions with TGF-β isoforms

A number of studies have linked S1Ps fibrotic activity with TGF-β1. In this study, we investigated the interplay between S1P/ SPHK I_2_ and the two TGF-β isoforms TGF-β1 and TGF–β3. In our previous studies, as well as others, have shown that TGF-β3 has antifibrotic properties [44].

**Fig 6:**
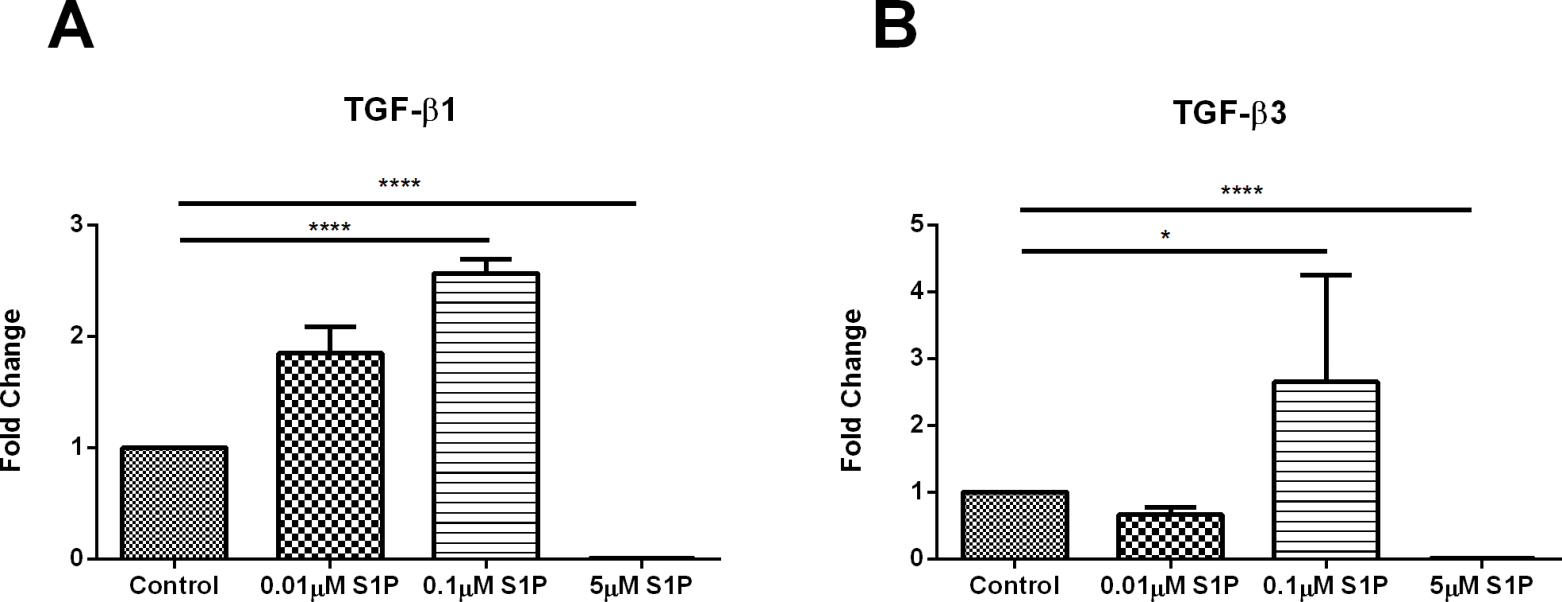
S1P and SPHK I_2_ signaling interactions with TGF-β isoforms/Quantification of western blots of HCF cell lysates following four week treatments with S1P or SPHK I_2_. (A) Treatment with 0.1μM concentration of S1P on HCF 3D constructs led to significant upregulation (p ≤ 0.0001) of TGF-β1 when compared to controls, where 5μM concentration of S1P on HCF 3D constructs led to significant upregulation (p ≤ 0.0001) of TGF-β1 when compared to controls. (B) Treatment with 0.1μM concentration of S1P on HCF 3D constructs led to significant upregulation (p ≤ 0.05) of TGF-β3 when compared to controls, where 5μM concentration of S1P on HCF 3D constructs led to significant upregulation (p ≤ 0.0001) of TGF-β3 when compared to controls. A one-way ANOVA was performed and P ≤ 0.05 considered statistically significant. * represents p ≤ 0.05, ** represents p ≤ 0.01, *** represents p ≤ 0.001, **** represents p ≤ 0.0001.

Fig 6 (S4 Fig) shows similar results when comparing TGF-β1 and TGF-β3 expression, following exogenous S1P stimulation. TGF-β1 showed significant (p≤0.01) upregulation following S1P stimulation at 0.1μM concentration, where significant (p≤0.0001) downregulation was observed at 5μM concentration (Fig 6A). Similarly, TGF-β3 expression was significantly (p≤0.01) upregulated at 0.1μM S1P and significantly (p≤0.0001) downregulated at 5μM S1P concentration (Fig 6B). TGF-β1 and TGF-β3 expression did not change following exogenous stimulation with SPHK I_2_.

When TGF-β1 was compared directly to TGF-β3 expression, following S1P stimulation, we found that only at 5μM S1P there was a significant (p≤0.0001) downregulation in TGF-β3 when compared to TGF-β1. In a similar comparison, for the SPHK I_2_ stimulated constructs, no differences were observed between TGF-β1 and TGF-β3 at any of the concentrations tested here.

## Discussion and conclusions

Human corneal fibrosis is a usual outcome following corneal injury. Despite significant advancements in the field, current treatments are limited. One of the problems is that corneal wound healing is a complex process, involving corneal cells, ECM components, and growth factors. The mechanisms by which corneal fibrosis can be prevented, or even reversed, remains elusive. The depth of the problem is clear when we consider that a total of 250 million people worldwide have compromised vision and around 6 million have been blinded, majorly due to corneal fibrosis and scarring.

The main characteristic of a corneal scar is the presence of myofibroblasts, often indicated by the expression of α-SMA, and the excessive and improper deposition of ECM components such as collagen III. Preventing scar formation would be ideal; however, studies investigating the development of non-fibrotic healing in human corneas are extremely limited. There are two main reasons for this: 1) Unavailability of human tissue makes investigations difficult, and 2) Mechanistic pathways of corneal scarring are not fully understood.

In this study, we investigated the role of SPLs in corneal fibrosis, using an *in vitro* model, and their interactions with a major player in the human cornea; TGF- β. SPLs have been recently linked to fibrosis in various of tissues and they are found to be in close connection to the TGF-β pathway. TGF-β is a superfamily of cytokines that exist as five isoforms, three of which are found in humans: TGF-β1, TGF-β2, and TGF-β3. The three isoforms have shown to affect a variety of biological processes which include cellular proliferation, differentiation, and migration in most cell types. In our previous studies, we found that while TGF-β1 exhibited profibrotic effects in HCFs, TGF-β3 displayed anti-fibrotic capabilities. Most notably, TGF-β3 was able to stimulate HCFs to secrete larger amounts of ECM, while maintaining anti-fibrotic characteristics, and exhibited a “rescuing” effect *in vitro* [57].

The cross-talk between S1P and TGFβ has been appreciated through several recent studies [10,13,58]. Briefly, it has been shown that TGFβ up-regulates *Sphk1* mRNA and protein levels and increases SPHK1 activity in dermal fibroblasts [59]. In contrast, TGFβ reduced S1P phosphatase activity. Interestingly, down-regulating *Sphk1* expression blocked TGFβ-mediated increases in TIMP-1 protein. It was therefore proposed that SPHK1 is a downstream mediator for the induction of TIMP1 by TGFβ [59]. It has also been demonstrated that S1P utilizes its receptor signaling to stimulate phosphorylation and activate TGFβRI kinase, resulting in phosphorylation of Smad2 and Smad3, independently of the TGFβ ligand, leading to keratinocyte proliferation and migration [23]. S1P-stimulated Smad phosphorylation was inhibited by down-regulation of TGFβ receptor type II and also by the S1P_3_ antagonist, suramin, suggesting that S1P transactivates TGFβRII in an S1P_3_-dependent process [22]. Consistent with these observations, another study showed that matrix remodeling in response to acute liver injury was abrogated in *S1P*_*2*_^*-/-*^ mice, which was attributed to reduced accumulation of myofibroblasts, as shown by a lower induction of a-SMA, TIMP1, TGFβ, and PDGF, suggesting that a-SMA induction by S1P may be S1P_2_- and Rho-dependent [60].

In the cornea, TGF-β is well studied whereas very little is currently known about the effects of S1P and its interrelations with the TGF-β pathway. Our study suggests that exogenous supply of S1P to HCFs downregulate SphK1, SphK2, and S1PR3 and had an effect on assembly by downregulating collagens I, V, and I/V ratio. Within the healthy cornea, the stromal ECM is composed primarily of Collagen I and Collagen V in a ratio of 80:20. This balance is critical for maintaining ECM integrity and regulating intracellular processes. The alteration seen here in Collagen I/III and I/V ratios, following S1P and SPHK I_2_ stimulation, suggests that modulating S1P/ SPHK I_2_ signaling may be critical for the balance and state of the corneal stroma ECM.

Furthermore, we observed downregulation of α-SMA and decreased cellular migration, which could indicate that S1P may have anti-fibrotic capabilities in the human cornea. S1P had the same effect on TGF-β1 and TGF-β3, where we observed upregulation with the 0.1μM concentration but downregulation with the 5μM concentration. Additionally, SPHK I_2_ seems to inhibit endogenous sources of S1P within the cell which signal for cellular actin mobilization thus causing decreased cellular migration. Our current findings suggest that S1P likely play important roles as both primary and secondary signaling messengers in the human cornea. Exogenous S1P is known to act as an important second messenger inside the cell [61] but most of its influences on cell signaling are reportedly due to cell surface receptor binding [45,62–64].

**Fig 7:**
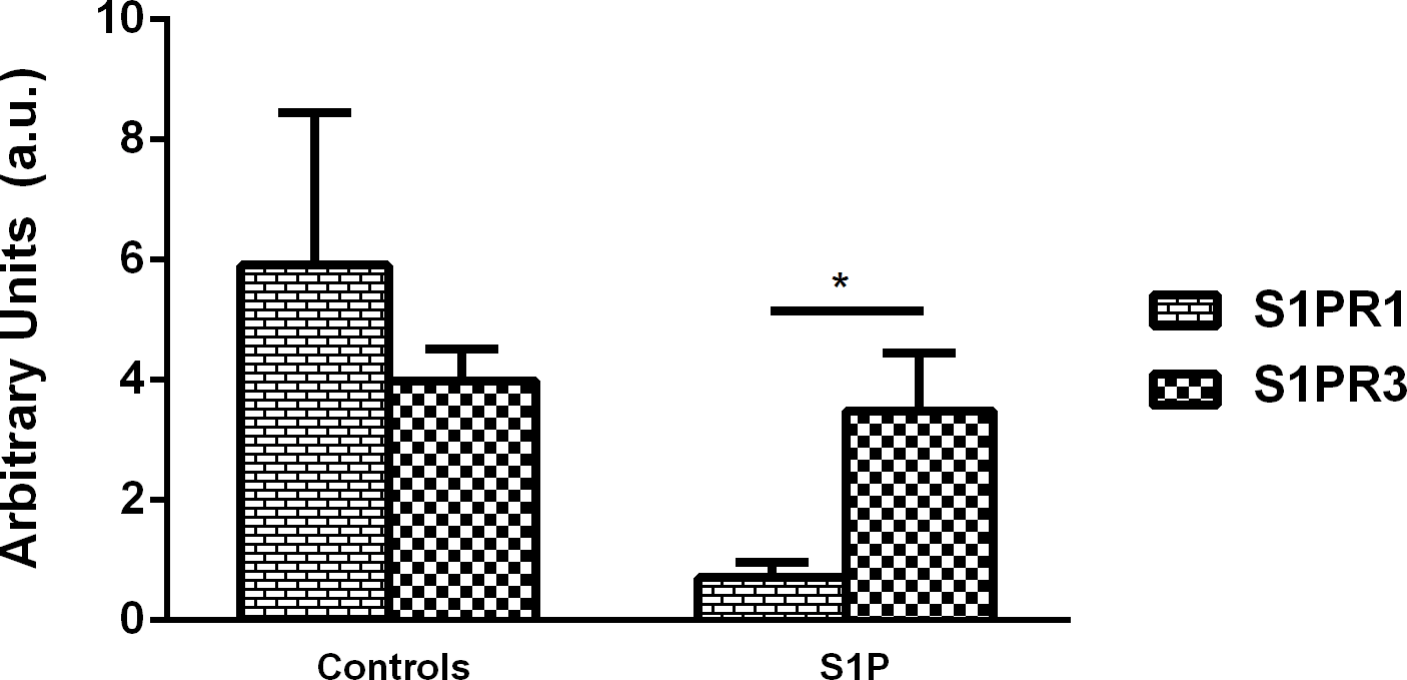
Effects of S1P on S1P receptors/Quantification of RT-PCR of HCF cell lysates following four week treatments with S1P. Treatment with 5μM concentration of S1P on HCF 3D constructs led to significant downregulation (p ≤ 0.0001) of S1PR1 when compared to S1PR3. A one-way ANOVA was performed and p ≤ 0.05 considered statistically significant. * represents p ≤ 0.05, ** represents p ≤ 0.01, *** represents p ≤ 0.001, **** represents p ≤ 0.0001.

Stimulation with S1P appears to induce or inhibit cell migration based on the tissue and cell type (cell-type specific). In our study, we observed that exogenous stimulation with S1P inhibits corneal cell migration and we suggest that S1P is acting through S1PR3 (Fig 7). It is reported in the following studies that S1P induced cell migration is mediated through S1PR1 and S1PR3. Our data revealed that S1P significantly downregulated S1PR1 and S1PR3 mRNA expression (Fig 7; p ≤ 0.05), further indicating that S1P inhibits cell migration.

Similarly, Wendler and co-authors observed reduced cell migration and inhibited mesenchymal cell formation in AV canal cushion tissue with increasing concentrations of S1P treatment. They suggested S1P is necessary for early heart development [45]. Becciolini et al. 2006 reported the anti-migratory actions of S1P in C2C12 myoblasts; stating that S1P inhibited cell migration and abolished chemotactic responses to IGF-1[65]. Arikawa et al. 2003 investigated the effects of S1P on migration of B16 melanoma cells. They found that S1P treatment led to inhibited migration of S1PR1 and S1PR3-overexpressing cells. They believe that their data indicates that S1PR2 mediates S1P inhibition of migration, whereas S1PR1 and S1PR3 mediate S1P stimulation of cell migration [66]. Kawa et al. 1997 found that S1P decreased trans-endothelial migration and invasiveness of neutrophils into human umbilical vein endothelial cells (HUVEC)-covered collagen layers. They suggest that S1P acts as a specific and effective motility regulator that could be used to mediate invasive migration of neutrophils [67].

Contrastingly, Simón MV et al. 2015 [35] observed that treatment with S1P enhanced Müller glial cell migration where SPHK I_2_ nearly prevented glial migration. They suggest that S1P is synthesized by Müller glial cells, which induces glial migration through S1PR3. Esche et al. 2010 also investigated the effects of S1P on Müller glial cell migration and observed slightly induced migration [68]. Li et al. 2011 examined the effects of S1P in the human hepatic myofibroblasts (hMFs) and stated that S1P displayed powerful migratory action on hMFs where S1PR1 and S1PR3 were significantly induced and S1PR2 was significantly inhibited [26]. Liu et al. 2011 investigated the functions of S1P in human hepatic stellate cells (HSC) line, LX-2 cells and found that S1P displayed powerful migratory action on LX-2 cells; S1PR1 and S1PR3 inducing migration and S1PR2 inhibiting migration [29]. Maceyka et al. 2008 determined that S1P induced cell migration through S1PR1 in human embryonic kidney cells (HEK 293), transfected with siRNA targeted to SphK [49]. Kimura et al. 2000 found that S1P induced both proliferation and migration on human aortic endothelial cells [51]. Previous studies also report the stimulation of endothelial cell migration by S1P [69–74].

Van Brocklyn, JR, 2010 published a review on the effects of S1P on cancer cell migration. He states that the effects of S1P on cell migration vary between different types of cancer and even within different models of the same cancer types, in some cases inducing migration and inhibiting in others [75]. The literature supports that some S1P receptors have opposite effects on cells/tissues [76]. S1PR2 has been reported to have anti-migratory effects whereas; S1PR1 and S1PR3 are reportedly pro-migratory in B16 melanoma cells [66], also in Chinese hamster ovary cells [77], human umbilical vein endothelial cells[78], mouse embryonic endothelial cells (MEECs)[79], bovine aortic endothelial cells (BAECs) [80], human gastric cancer cell lines [81], and in human hepatic myofibroblasts[26] (hMFs).

Kimura et al. 2000 and Wendler C, Rivkees SA, 2006 report oppositional effects of S1P in the heart as we’ve observed in the eye; oppositional effects in the human cornea and in the retina. Means CK, Brown JH, 2009 published a review on S1P receptor signaling in the heart where they mention, based on the literature, it is known that S1P inhibits migration of smooth muscle cells where it induces migration of endothelial cells [82,83]. These findings are very interesting as our study suggests that S1P inhibits cell migration in HCFs whereas it promotes migration in the retina [35]. The eye, like the heart, is a complex organ with various cell types that appear to respond differently to S1P. As we have just scratched the surface of S1P signaling in the cornea, further investigations into the downstream signaling effects and interplay with TGF-β are necessary for the future potential design of novel therapeutics targeting S1P signaling in the opportune timeframe post incidence of corneal fibrosis to successfully manage ECM remodeling. In conclusion, the role of S1P in corneal biology is still unclear. Further elucidation of how S1P modulates TGF-β signaling may lead to the discovery of pro-fibrotic pathways that may be altered to reduce corneal fibrosis.

## Supporting information

S1 Fig Western blots representing the effects of S1P and SPHK I_2_ on SphK1, SphK2, and S1PR3. Control, 0.01μM, and 0.1μM were run together on western blot gels, where 5μM (with controls) was run separately as indicated within the dotted lines. Controls are representative from both runs.
(PDF)

S2 Fig Western blots representing the effects of S1P and SPHK I_2_ on α-SMA. Control, 0.01μM, and 0.1μM were run together on western blot gels, where 5μM (with controls) was run separately as indicated within the dotted lines. Controls are representative from both runs.
(PDF)

S3 Fig Western blots representing the effects of S1P and SPHK I_2_ on Collagens I, V, and III. Control, 0.01μM, and 0.1μM were run together on western blot gels, where 5μM (with controls) was run separately as indicated within the dotted lines. Controls are representative from both runs.
(PDF)

S4 Fig Western blots representing the effects of S1P on TGF-β1 and TGF-β3. Control, 0.01μM, and 0.1μM were run together on western blot gels, where 5μM (with controls) was run separately as indicated within the dotted lines. Controls are representative from both runs.
(PDF)
