# Supplementary figures and images for: Unravelling the interplay of sphingolipids and TGF-β signaling in the human corneal stroma
Source: PLoS One. 2017 Aug 14;12(8):e0182390. doi: 10.1371/journal.pone.0182390 (PMC5555661; doi:10.1371/journal.pone.0182390)

### S1P stimulation

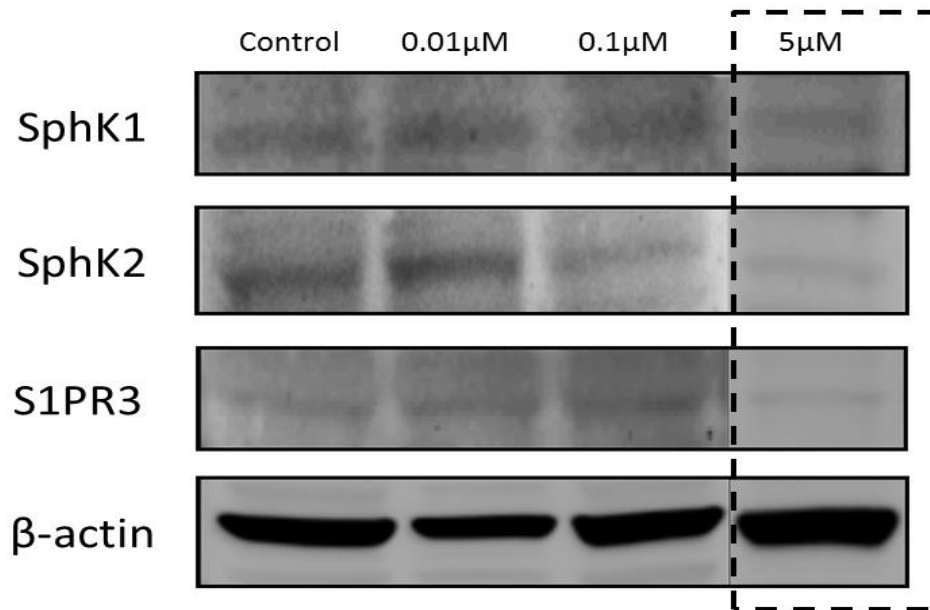

### SPHK I<sub>2</sub> stimulation

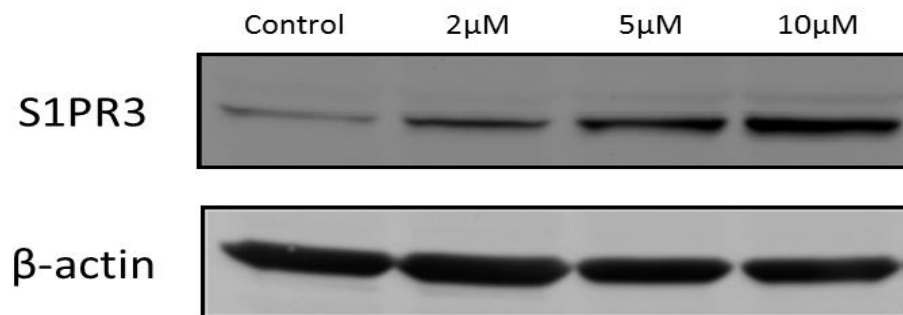

Supplement: S1 Fig — Control, 0.01μM, and 0.1μM were run together on western blot gels, where 5μM (with controls) was run separately as indicated within the dotted lines. Controls are representative from both runs. (PDF) [file pone.0182390.s001.pdf]

### S1P stimulation

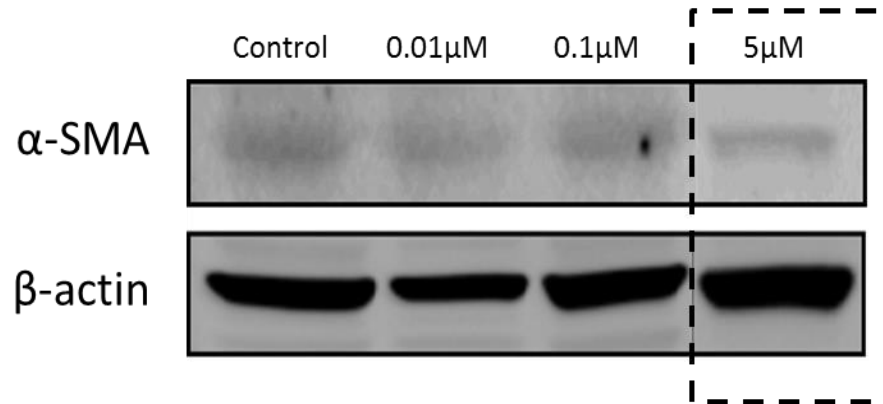

### SPHK I<sub>2</sub> stimulation

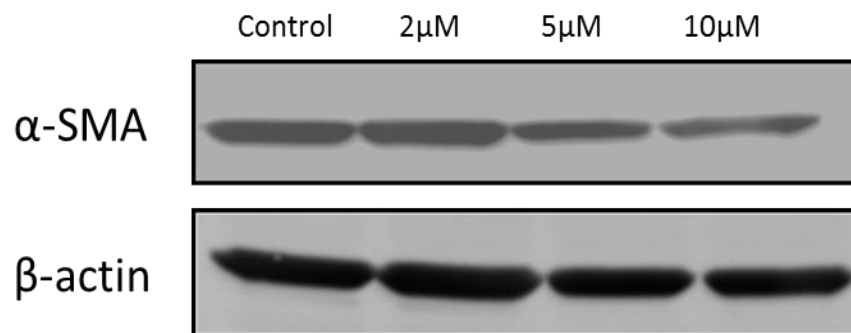

Supplement: S2 Fig — Control, 0.01μM, and 0.1μM were run together on western blot gels, where 5μM (with controls) was run separately as indicated within the dotted lines. Controls are representative from both runs. (PDF) [file pone.0182390.s002.pdf]

### S1P stimulation

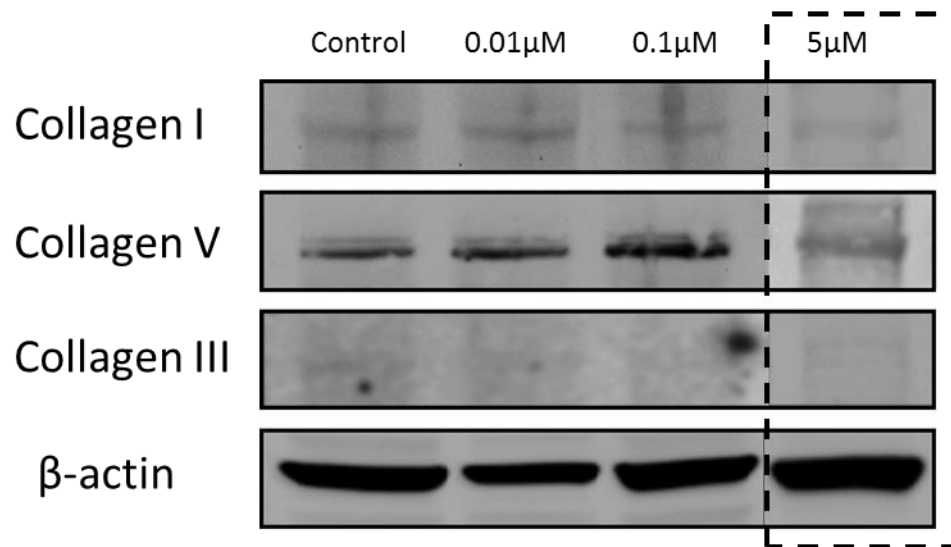

### SPHK I<sub>2</sub> stimulation

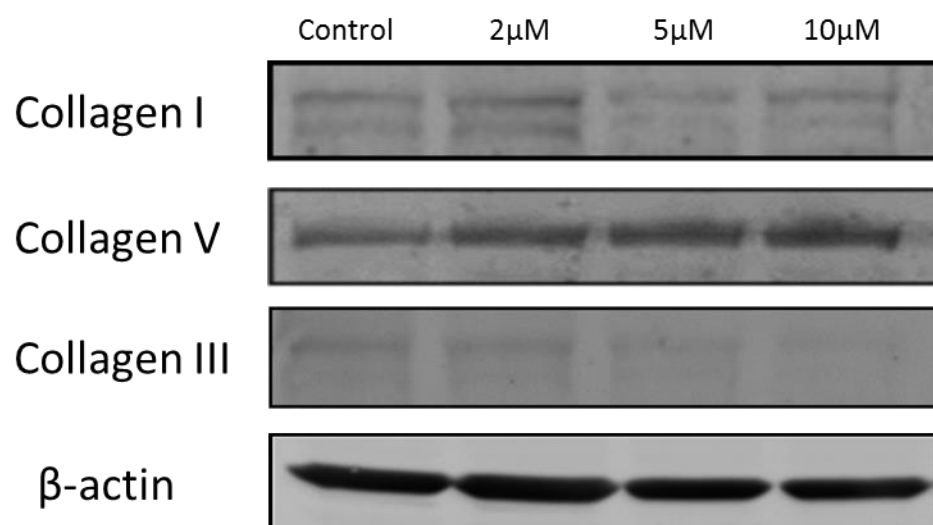

Supplement: S3 Fig — Control, 0.01μM, and 0.1μM were run together on western blot gels, where 5μM (with controls) was run separately as indicated within the dotted lines. Controls are representative from both runs. (PDF) [file pone.0182390.s003.pdf]

### S1P stimulation

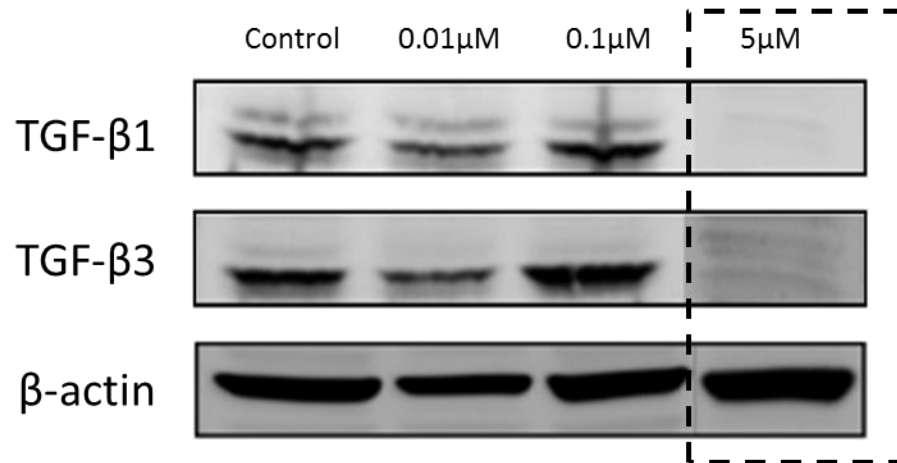

Supplement: S4 Fig — Control, 0.01μM, and 0.1μM were run together on western blot gels, where 5μM (with controls) was run separately as indicated within the dotted lines. Controls are representative from both runs. (PDF) [file pone.0182390.s004.pdf]
